# Cascading effects of artificial light at night: resource-mediated control of herbivores in a grassland ecosystem

**DOI:** 10.1098/rstb.2014.0131

**Published:** 2015-05-05

**Authors:** Jonathan Bennie, Thomas W. Davies, David Cruse, Richard Inger, Kevin J. Gaston

**Affiliations:** Environment and Sustainability Institute, University of Exeter, Penryn TR10 9FE, UK

**Keywords:** light pollution, photopollution, artificial light at night, biotic interactions, community-level, bottom-up effects

## Abstract

Artificial light at night has a wide range of biological effects on both plants and animals. Here, we review mechanisms by which artificial light at night may restructure ecological communities by modifying the interactions between species. Such mechanisms may be top-down (predator, parasite or grazer controlled), bottom-up (resource-controlled) or involve non-trophic processes, such as pollination, seed dispersal or competition. We present results from an experiment investigating both top-down and bottom-up effects of artificial light at night on the population density of pea aphids *Acyrthosiphon pisum* in a diverse artificial grassland community in the presence and absence of predators and under low-level light of different spectral composition. We found no evidence for top-down control of *A. pisum* in this system, but did find evidence for bottom-up effects mediated through the impact of light on flower head density in a leguminous food plant. These results suggest that physiological effects of light on a plant species within a diverse plant community can have detectable demographic effects on a specialist herbivore.

## 1. Background

Light is a major abiotic force influencing the physiology, behaviour and reproduction of both plants and animals. Disruption of natural cycles of light by the introduction of artificial light at night has been shown to have marked effects on many species by altering their physiology or behaviour [1–3]. It is likely that these impacts in turn alter rates of resource use, reproduction, mortality, immigration and emigration at the level of populations [4] and may result in changes in patterns of abundance and the distribution of species, the structuring of ecological communities and in the functioning of ecosystems [5,6]. To date, few studies have explicitly addressed these concerns and sought to document whether such changes take place, what form they take and how severe they can be (but see [5,7]).

Nevertheless, the global extent of artificial light at night raises concern that such ecological impacts may be widespread. The responses of natural communities to anthropogenic pressures on the environment are often mediated by interactions between species [8]. Direct effects on a single species can lead to complex and far-reaching indirect effects on others and on the structure and function of the ecosystem. Examples of such cascading effects exist across marine, freshwater and terrestrial ecosystems [9–13]. Such biotic forces are often characterized as top-down (predator or parasitoid controlled) or bottom-up (resource-controlled) and may involve multiple trophic links [14–16]. Non-trophic interactions between species, such as pollination and seed dispersal [17,18], and competition [19], also have the potential to restructure ecological communities. It is widely recognized that both top-down and bottom-up controls occur in many systems [20] and that the relative influence of these controls can be context-dependent, varying with season [21,22], location [23,24] and ecosystem type [25]. Both bottom-up and top-down regulation may be triggered by changes in the behaviour [9,26], in physiology [27] and/or the population density, species composition or biomass within a trophic level [28]—and their effects may be detectable through changes at the community level in terms of shifts in species composition [29], or changes in the density or abundance of another species or group [30].

**Figure 1.**
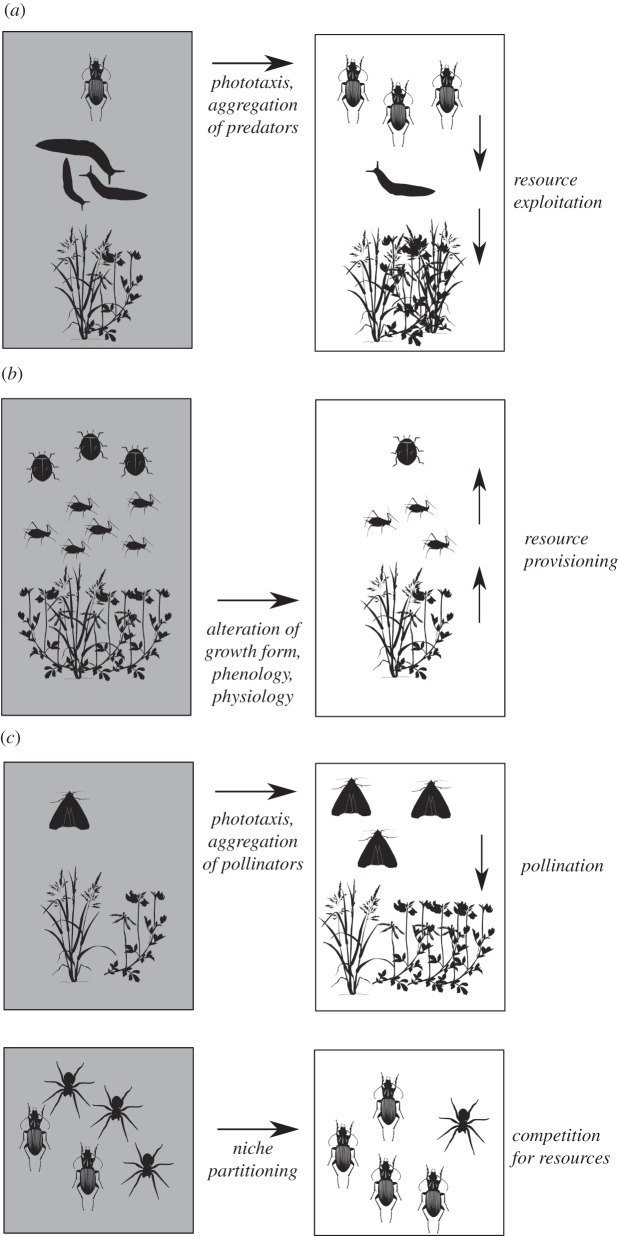
Some potential impacts of artificial night-time light on a grassland ecosystem. (*a*) Top-down trophic effects may occur if aggregation, population growth or greater foraging efficiency of consumers leads to higher resource exploitation, and potential cascading effects to lower trophic levels. (*b*) Bottom-up trophic effects may occur if light-induced changes in the physiology, abundance or composition of primary producers (plants) alter the provisioning of resources to higher trophic levels. (*c*) Non-trophic effects may occur if other links between species—such as pollination or competition within a trophic level—are modified by artificial light.

Many anthropogenic pressures on the environment affect primarily top-down or bottom-up processes—for example, the removal or introduction of top predators [13] or nutrient inputs to vegetation [31]. Other pressures, such as climate change, have the potential to influence top-down, bottom-up and non-trophic interactions simultaneously [32,33]. Artificial light at night likely falls into this latter category as direct impacts of light are widespread across groups [1,2]. Detailed, universally applicable predictions as to how indirect effects will manifest are challenging to make, because the strength of interactions varies with context. Here, we first review what is known of the effects of artificial light at night on top-down, bottom-up and non-trophic interactions. We then investigate its potential for indirect, biotic effects (top-down and bottom-up) on ecosystem structure and function using a temperate grassland assemblage as a model ecosystem (figure 1). Our findings demonstrate that artificial light controls the abundance of a specialist herbivore indirectly by influencing flowering (and so resource availability) in a leguminous plant. The effects of artificial light at night on grassland systems is non-trivial, particularly given the importance of roadside grassland vegetation as a conservation resource in the agricultural landscapes of temperate regions [34–37], and the increasing spread of artificial lighting of roadside verges by street lighting [38].

### (a) Top-down effects

Changes in the abundance of parasites, predators and herbivores can change the populations of hosts, prey species and primary producers through top-down effects [39–41]. Aggregation of predators has been shown to be able to affect vegetation biomass in grassland systems [39], and predator functional composition can influence plant community composition and ecosystem functions such as litter decomposition and nitrogen mineralization [42]. Many studies show that artificial light at night can affect the distribution, abundance, behaviour and activity patterns of predators and foragers in a number of ways. Light at night can cause aggregation or locally increased populations of predators around the light source [5,43–45]. Diurnal or crespuscular predators may extend their activity into the night [46–49], effectively increasing predation pressure. Some nocturnal predators increase prey detection and capture rates under low levels of artificial light [50,51]. By contrast, some nocturnal species may avoid lit areas or reduce activity under lit conditions [52]. Aggregation of predators can also cause a behavioural response in the form of predator avoidance at lower trophic levels [26].

In a study in roadside grassland, predatory carabid beetles have been observed in higher abundance under high-pressure sodium street lighting compared with darker patches between the lights [5]. Although the street lighting was not found to affect the abundance of any grazing taxa sampled, greater numbers of predatory individuals suggests ramifications for the activity and/or density of prey species, and potentially on the plants on which they feed (figure 1*a*).

### (b) Bottom-up effects

Artificial light at night can impact directly on plants [53,54]. In addition to conversion of sunlight into energy via photosynthesis (to which the relatively low light levels experienced by plants under artificial light at night probably make a very minor contribution), plants respond to their natural light environment through photoreceptors, the best understood of which are the phytochrome family. Phytochrome has several physiological roles and is used by plants to receive information concerning time of year (day length) and shading by other plants and to trigger responses in terms of germination, vegetative development and phenology [55,56] and growth form, particularly allocation to reproductive and vegetative growth [57]. Phytochrome exists in two interchangeable forms, Pr, which preferentially absorbs light in the red portion of the spectrum, and Pfr, which preferentially absorbs in the far-red. The ratio between red and far-red wavelengths of light in particular is thus detected by phytochrome and is used by plants to infer information about their environment. Hence red lights are used in horticulture to control flowering and shoot elongation [58,59]. Even low levels of light typical of street lighting, and/or brief periods of exposure during the hours of darkness, are often sufficient to produce a response [54]. Street lighting has long been observed to alter the phenology of urban trees [54,60], can delay, inhibit, advance or promote flowering [53] and may even alter the flowering and vegetative growth of crops [54,61]. If such effects are widespread in natural and semi-natural vegetation under artificial lighting, they may lead to bottom-up, resource-mediated effects (figure 1*b*) on herbivores. Bottom-up effects may be driven not only by the quantity of resources for herbivores and detritivores [62], but also by restructuring habitat and affecting the availability, quality and diversity of food resources [63,64]. Bottom-up effects can span several trophic levels—for example, Koricheva *et al.* [30] found that grassland plant diversity affected not only herbivore populations (particularly specialist and sessile herbivores, such as wingless aphids) but also influenced predator activity.

### (c) Non-trophic interactions

Perhaps the most well-known environmental effect of artificial light at night is the attraction of moths and other aerial invertebrates [44,65–69]. The behaviour of bats is also strongly influenced by artificial light [45,70–74]. Both groups can be important nocturnal pollinators of plants, and frugivorous bats can be important seed dispersers. The effect of artificial light on pollinating and seed dispersing species could result in reduced or enhanced recruitment among plants leading to changes in vegetation composition (figure 1*c*) [75], but with few exceptions [6] the effects of artificial lights on these ecosystem services has not been studied.

Competition between species for resources is another biotic interaction that may be influenced by artificial light at night. Artificial light at night can itself be viewed as a resource [2] and expand or restrict the availability of time for activities such as hunting or foraging. Species have evolved to differentiate their activity time along temporal gradients of daylight and darkness giving rise to niche partitioning of the 24 h cycle [76,77]. Artificial light may alter the balance between species in favour of those that are able to use the ‘night light niche’ [46,49,77] with indirect consequences for their competitors.

### (d) Testing for trophic effects

Here, we report data from an experimental study of artificially assembled plant and invertebrate communities under realistic conditions. We test whether artificial light at night affects population densities of a specialist herbivore, the pea aphid *Acyrthosiphon pisum*, and whether these effects are mediated by (i) top-down processes of exploitation, by controlling the presence or absence of predators, the ladybird *Adalia bipunctata* and carabid beetle *Pterostichus melanarius*; or (ii) bottom-up processes, by measuring the availability of a resource, the flowering shoots of the leguminous plant *Lotus pedunculatus*. Several feasible pathways exist for effects of artificial light on this system. Day length is known to be critical in the control of flowering in *L. pedunculatus* [78], and thus artificial light could alter the availability of resources for *A. pisum*. Both predator species in the experiment use visual cues to locate prey and therefore light may affect their behaviour [79,80]; in other ladybird species, photoperiod and wavelength of light affect reproductive performance [81]. Finally, photoperiod has direct effects on reproduction in *A. pisum* itself [82]. We found no evidence for top-down predator-mediated control of *A. pisum* in our system, but did find evidence for bottom-up effects, demonstrating that physiological effects of light on a plant species within a diverse plant community can have detectable demographic effects on a specialist herbivore.

## 2. Material and methods

**Figure 2.**
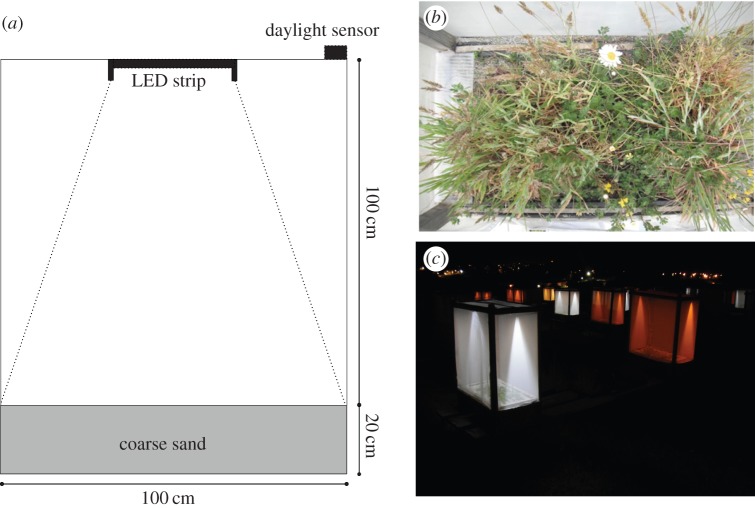
(*a*) Elevation view of a mesocosm, showing sand substrate and mesh cage with LED strip controlled by a daylight sensor on top of the cage. (*b*) Example of vegetation within a mesocosm in July 2013. (*c*) Photograph of the experiment at night.

The data presented here are generated from the first year of a long-term experiment to examine the effects of artificial light at night on trophic interactions in model grassland ecosystems. Fifty-four experimental grassland ‘mesocosms' were established outdoors in July 2012 at the University of Exeter's Penryn Campus (50°10′ N, 5°7′ W). Each mesocosm consists of a 1 m × 0.5 m × 0.2 m trough, lined with woven plastic textile for drainage and filled with coarse builder's sand, and mounted on wooden planks 0.75 m above the ground (figure 2). A wooden frame 1 m tall and lined with fine anti-thrip mesh, with a zip for access for maintenance and measurements, was mounted on top of the trough to isolate the invertebrate community. Seventy-two individual plants, representing four individuals from each of 18 common grassland species (grown in spring from seed gathered from wild plants in 2011) were planted in a randomized grid pattern 5 cm apart within the central section of each mesocosm in July 2012 (see the electronic supplementary material for details of plant species). A standard nutrient solution was applied to each mesocosm during July 2012 to establish initial plant growth.

Two different artificial light treatments were applied to mesocosms, each consisting of a strip of light-emitting diodes (LEDs) mounted on a wooden bar across the top of the mesocosm and facing downwards. The ‘white’ treatment consisted of ‘cool white’ LEDs, with a spectrum similar to those in commercial LED street lighting systems, a peak in the blue portion of the spectrum (around 445 nm) and a broad secondary peak between around 500 and 650 nm. The stated correlated colour temperature (CCT) of the white LED strip is 6000 K (stated here for comparison with other white LED sources—it should be stressed that the CCT of light sources that are not black-body emitters is an estimation of their appearance to human vision and is a poor description of either the physical properties or biological effects of the light source [83]). The ‘amber’ treatment consisted of a virtually monochromatic LED strip with a single narrow peak in the orange portion of the spectrum, at around 588 nm, aiming to simulate the peak emittance of monochromatic low-pressure sodium (LPS) lighting (589.3 nm), which was formerly in widespread use in the UK and elsewhere, and is still the most common form of lighting in many regions. LED lights were used to simulate LPS lighting as unlike LPS gas-discharge lamps they emit negligible heat and would not affect the temperature of the mesocosms. The LED strips were cut to a length so that both lighting treatments provide an illuminance of approximately 10 lx at the unshaded sand surface and 15 lx at 20 cm height at a perceived brightness similar to human vision to that measured on a lit grassland road verge under street lights [84,85]. Light treatments are powered by a 12-V battery and triggered by light-detecting photocells mounted on top of the frame, to switch on at sunset (less than 70 lx) and off at sunrise (more than 110 lx). In addition, unlit ‘control’ treatments reproduced the mounting bar and structure of the lit treatments but had no LED strips.

These three light treatments (white, amber and control) were implemented in a cross factorial design with three levels of community complexity—plants only, bitrophic and tritrophic, with each light-trophic treatment combination replicated six times. The plants-only treatments contained the grassland plant species and were treated at regular intervals with a biodegradable insecticide (pyrethrin) and molluscicide (ferric phosphate pellets) to prevent the establishment of invertebrate populations. Both the bitrophic and tritrophic treatments received introductions of 20 individuals each of the pea aphid *A. pisum* and the slug *Deroceras reticulatum* in May and June 2013. In addition, the tritrophic treatments received introductions of four individuals (one male, three females) of the predatory ground beetle *P. melanarius* in August 2013 and nine unsexed individuals of the ladybird *A. bipunctata* in May to June of 2013. Six replicates of each light and trophic treatment combination (nine possible light/trophic combinations) were allocated randomly to mesocosms evenly distributed within a field, separated from each other by at least 4 m and offset to minimize light spillage between mesocosms. Typical recorded ambient light levels at full moon at the site were around 0.11 lx under clear sky conditions and 0.04 lx when the moon was obscured by cloud; due to the proximity of the site to a university campus and suburban areas, some degree of skyglow from the surrounding area is expected. To the level of detection of the light meter (±0.01 lx), light levels measured in control mesocosms at night did not differ measureably from ambient levels measured outside at the site with treatments switched off.

Here, we present data on the density of inflorescences of the leguminous plant *L. pedunculatus*, within the mesocosms and the observed population density of *A. pisum*, which is a specialist herbivore of legumes and was found almost exclusively on *L. pedunculatus* in the mesocosms. Exhaustive counts of the number of flower heads of each species (classified into three phenological classes) and 3-min timed counts of aphids within each mesocosm were carried out at bi-weekly intervals from April to September. It was not possible systematically to monitor the populations of predators within the mesocosms due to difficulty in locating the animals within the dense vegetation; however, individuals of both species were recorded within the tritrophic treatments throughout the study period, suggesting that populations were maintained for the duration of the results reported here.

Inflorescence and aphid count data were analysed separately using general linear mixed effects models using glmmADMB [86] in the R statistical package (v. 3.1.0; R Core Team 2014) with light treatment, survey date and trophic treatment (presence or absence of predators for both aphid and inflorescence counts, and presence or absence of herbivores for inflorescence counts) included as fixed factors, and plot identity as a random effect (intercept) to allow for repeated measurements at the same plot at different time steps. Poisson, negative binomial, zero-inflated Poisson and zero-inflated negative binomial (ZINB) models were fitted to each dataset. Alternative models consisting of full combinations of fixed factors and interaction terms were tested, and significance values are reported here for the best model in each case, with model quality assessed by Akaike information criterion (AIC) values (electronic supplementary material).

## 3. Results

*Lotus pedunculatus* inflorescence counts were best characterized by a repeated measures ZINB model incorporating interacting effects of time and herbivory (but not presence of predators) and an effect of light treatment. The presence of herbivores and both the amber and white light treatments significantly decreased flower density in *L. pedunculatus* relative to controls with a greater effect in the amber treatment (*p* = 0.035 for herbivory, *p* = 0.002 for amber light and *p* = 0.042 for white light). The addition of the presence or absence of predators as a fixed factor, or further interactions between light treatments, herbivory and survey date gave no additional explanatory power to the model (see the electronic supplementary material).

**Figure 3.**
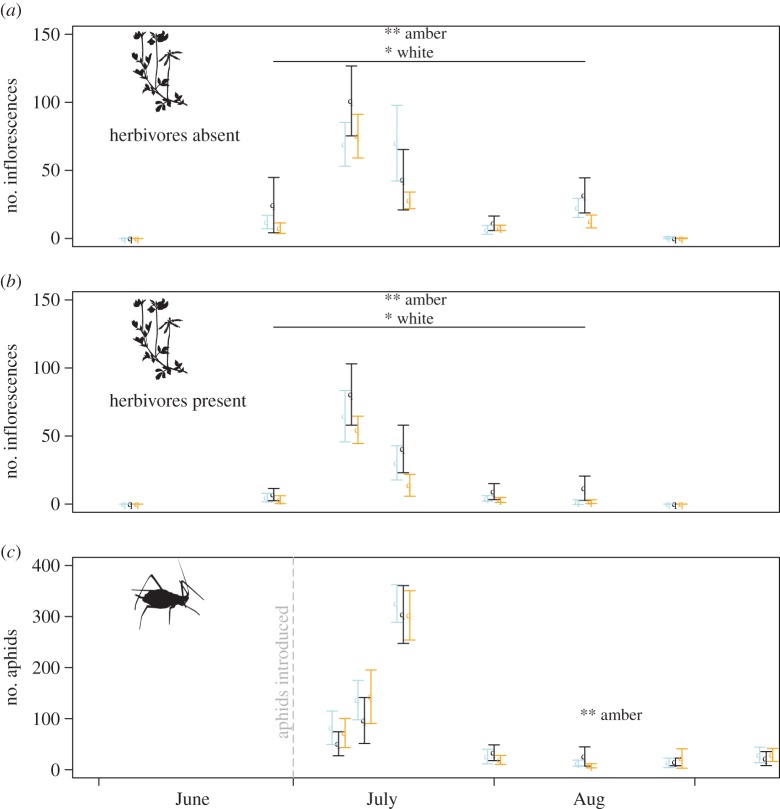
(*a*,*b*) Counts of flower heads of the leguminous plant *L. pedunculatus* in experimental grassland mesocosms in summer 2013, in the presence and absence of herbivores. Black shows controls (unlit at night), blue shows white light treatment and orange lines show amber light treatment (mean ± s.e.). (*c*) Three-minute timed counts of pea aphids *A. pisum* in mesocosms in 2013, colours as above. Asterisks represent significant differences between the light treatment and control—for *L. pedunculatus* inflorescence counts were consistently lower under both light types during the flowering season, across all survey dates and independent of the presence of herbivores. For *A. pisum*, counts were significantly lower under amber lights only during mid-August. At this time, *A. pisum* was found almost exclusively on *L. pedunclatus* flowers, which were rare in the lit treatments.

*Acyrthosiphon pisum* counts were best characterized by a repeated measures negative binomial model incorporating interactions between survey date and light treatment; again the addition of the presence or absence of predators as a factor did not increase model parsimony. The interaction term between light and survey date showed a significant decrease in aphid numbers under amber lights only in mid-August (*p* = 0.006), after peak flowering of *Lotus* (figure 3); at the same date, the aphid counts under white light were lower, but not significantly so at a 95% significance threshold (*p* = 0.072).

## 4. Discussion

The mesocosm experiment provides evidence for a potential ‘bottom-up’ mechanism by which artificial night-time light at levels typical around human settlements can both directly affect growth form and reproductive effort in plants, and indirectly affect herbivore density. Flowering in *L. pedunculatus* plants in artificial grassland communities peaked in early July 2013 and fell in late July and August, partly due to a period of dry weather. Unsurprisingly, the presence of herbivores suppressed the density of flower heads throughout the year, but in both the presence and absence of herbivores flowering was also suppressed by monochromatic amber light at night at a peak wavelength similar to LPS street lighting and to a slightly lesser extent by white LED lighting. Previous studies have shown that *L. pedunculatus* is a long-day plant, and individuals of northern European origin failed to produce flowers when introduced to lower latitudes in northern New Zealand where summer days are not sufficiently long [78]. However, links between spectra, intensity and physiological triggers can be complex [54] and in this experiment low intensity light appears to inhibit, rather than induce flowering.

The number of aphids recorded showed no effect of light in July, but showed significantly suppressed numbers under the amber light treatment in mid-August. In spring and early summer, *A. pisum* within the mesocosms fed mainly on vegetative shoots of *L. pedunculatus* and other legumes, but by August *L. pedunculatus* had effectively ceased vegetative growth, and flower heads and developing seed pods of this species provided the main source of nutrition for these sap-feeding insects. We conclude that the seasonal suppression of the aphid population under the amber light treatment in mid-August is most likely caused by resource limitation for this species due to suppression of flowering.

Although we were not able to test for a direct effect of light on predator numbers, we found neither an effect of predator presence/absence on aphid numbers, nor an interaction effect between predation and light treatment. The lack of explanatory power of predator presence in predicting *A. pisum* counts suggests that top-down effects of *A. bipunctata* on the density in this species were weak compared with bottom-up effects, and that aphid numbers were primarily resource-controlled.

**Figure 4.**
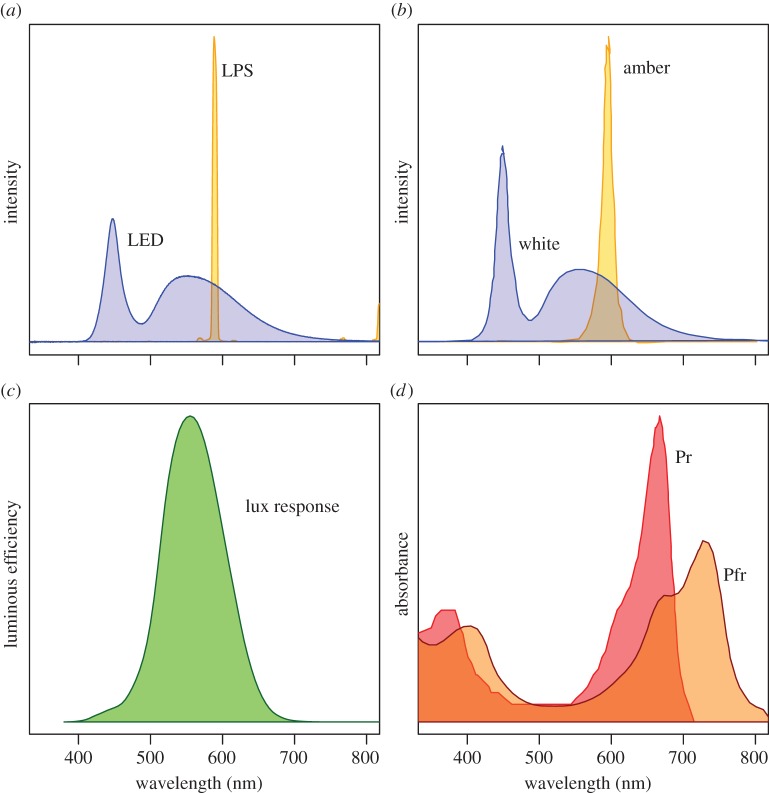
(*a*) Spectral distribution of light from two street light types; LPS lamps emit an almost monochromatic yellow-orange light, while commercial white LED lights typically emit a primary peak in the blue portion of the spectra with a wide secondary peak within human-visible wavelengths. Both spectra were measured at night in road verges under street lights in Cornwall, UK, using a spectrophotometer (Maya2000 pro, Ocean Optics, Dunedin, FL, USA). (*b*) Spectral distribution of light measured in the two experimental light treatments. (*c*) A lux response curve, an approximation of the photopic sensitivity of the human eye, widely used in the lighting industry to convert light intensity at different wavelengths into measures of perceived brightness (lux or lumens). (*d*) Absorbance spectra for the two forms of the plant pigment phytochrome, Pr (which preferentially absorbs red light) and Pfr (which absorbs far-red light).

The most significant effects detected here were shown for the ‘amber’ light treatment, which was designed to simulate traditional LPS lighting. Responses for the ‘white’ light treatment, with a spectral distribution similar to those of modern commercial LED street lighting, were intermediate between the amber and control treatments (figure 3). The clearer effects of amber light are consistent with the response of phytochrome to the ratio between photons absorbed by the Pr and Pfr forms (figure 4). The peak emittance at around 590 nm is absorbed preferentially by phytochrome in its Pr state, converting it to the physiologically active Pfr state. For a given perceived brightness (as measured in photometric units such as lux or lumens), the white LED treatment emits fewer photons within the peak absorbance of Pr. Much concern as to the health and environmental effects of artificial light focuses on the expansion of ‘whiter’ light across a wider range of wavelengths and on light produced at the lower end of the visible spectrum, largely because blue light (as produced by LEDs) controls melatonin levels and circadian rhythms in humans and other animals [87], and light at low wavelengths is more effective at attracting flying invertebrates [68]. However, this study demonstrates that artificial light at the higher wavelengths that plants respond to via the phytochrome pathway may have ecologically significant effects, not only on the physiology of plants themselves, but via bottom-up biotic interactions to animal populations.

## 5. Conclusion

Many direct effects of night-time artificial light on plant and animal species have been documented, and there is a growing body of evidence concerning the physiological and behavioural impacts of light pollution. However, it is unknown at present to what extent artificially lit ecosystems differ in their structure and dynamics to natural systems. There is a pressing need for an understanding of the ecosystem level effects of light pollution. This study found evidence for bottom-up control of a sessile, specialist herbivore, mediated by control of flowering in its foodplant presumably through the phytochrome pathway. A near-monochromatic amber light source had a greater effect on this pathway than a white LED source at a similar luminous flux. However, there is clearly also scope for top-down and non-trophic effects of light in temperate grassland systems, with the relative strength of these pathways potentially dependent on the intensity, spectral composition and spatial pattern of light. Untangling the importance and scale of such effects will require both experimental manipulations and field observations.

## Supplementary Material

Supplementary data

## Supplementary Material

Supplementary material

## Data accessibility

The datasets presented in this paper are included in the electronic supplementary material.
