# Supplementary material for: Cascading effects of artificial light at night: resource-mediated control of herbivores in a grassland ecosystem
Source: Philos Trans R Soc Lond B Biol Sci. 2015 May 5;370(1667):20140131. doi: 10.1098/rstb.2014.0131 (PMC4375371; doi:10.1098/rstb.2014.0131)
Supplement: Supplementary material [file rstb20140131supp2.pdf]

Bennie *et al.* 2015 Cascading effects of artificial light at night: resource-mediated control of herbivores in a grassland ecosystem. *Phil. Trans. R. Soc. B.* doi: 10.1098/rstb.2014.0131

Supplementary material

List of plant species in mesocosms

Four individual plants of each of the following 18 species (total of 72 plants) were planted in a randomised grid.

Grasses
Anthoxanthum odoratum
Agrostis capillaris
Holcus lanatus
Cynosurus cristatus
Dactylis glomerata
Festuca ovina

Leguminous herbs
Lotus corniculatus
Lotus pedunculatus
Trifolium dubium
Trifolium pratense

Non-leguminous herbs
Leucanthemum vulgare
Achillea millefolium
Leontodon saxatilis
Hypochaeris radicata
Prunella vulgaris
Centaurea nigra
Ranunculus acris
Plantago lanceolata

© The Authors under the terms of the Creative Commons Attribution License http://creativecommons.org/licenses/by/3.0/, which permits unrestricted use, provided the original author and source are credited.

**Lotus pedunculatus inflorescences**		
**Fixed factors**	**AIC**	**ΔAIC**
Light+Date*Herbivores	1978.13	0
Light+Date+Herbivores	1984.50	6.37
Light+Date	1998.18	20.05
Light*Date*Predators+Date*Herbivores	1998.31	20.18
Null model	1999.96	21.83

**Acyrthosiphon pisum individuals**		
**Fixed factors**	**AIC**	**ΔAIC**
Light*Date	2460.80	0
Light*Date+Predators	2462.38	1.58
Light*Predators*Date	2467.96	7.16
Light*Date + Predators*Date	2472.50	11.70
Null model	2476.62	15.82

Supplementary Table 1: Description of the four models with the lowest AIC values explaining observed counts *of Lotus pedunculatus* flower heads and *Acyrthosiphon pisum* adults in 2013. All models contained plot (mesocosm) as a random intercept to allow for repeated measures. “Light” indicates light treatment (control, white or amber), “Herbivores” indicates presence or absence of herbivores, “Predators” indicates presence or absence of predators, “Date” indicates date of survey. ‘+’ between terms indicates non-interacting terms; ‘*’ indicates fully interacting terms. For L. pedunculatus we selected a zero-inflated negative binomial model, for *A. pisum* a negative binomial model without zero-inflation.

© The Authors under the terms of the Creative Commons Attribution License http://creativecommons.org/licenses/by/3.0/, which permits unrestricted use, provided the original author and source are credited.
